# Lipid Peroxidation via Regulating the Metabolism of Docosahexaenoic Acid and Arachidonic Acid in Autistic Behavioral Symptoms

**DOI:** 10.3390/cimb45110574

**Published:** 2023-11-15

**Authors:** Kunio Yui, George Imataka, Tadashi Shiohama

**Affiliations:** 1 Department of Pediatrics, Graduate School of Medicine, Chiba University, Chiba 260-8677, Japan; 2 Department of Urology, Fujita Health University School of Medicine, Toyoake 470-1192, Japan; 3 Department of Pediatrics, Dokkyo Medical University, Tochigi 321-0293, Japan

**Keywords:** lipid peroxidation, ferroptosis, malondialdehyde-modified low-density lipoprotein, autistic behavior, do cosahexaenoic acid, arachidonic acid

## Abstract

The association between the lipid peroxidation product malondialdehyde (MDA)-modified low-density lipoprotein (MDA-LDL) and the pathophysiology of autism spectrum disorder (ASD) is unclear. This association was studied in 17 children with ASD and seven age-matched controls regarding autistic behaviors. Behavioral symptoms were assessed using the Aberrant Behavior Checklist (ABC). To compensate for the small sample size, adaptive Lasso was used to increase the likelihood of accurate prediction, and a coefficient of variation was calculated for suitable variable selection. Plasma MDA-LDL levels were significantly increased, and plasma SOD levels were significantly decreased in addition to significantly increased plasma docosahexaenoic acid (DHA) levels and significantly decreased plasma arachidonic acid (ARA) levels in the 17 subjects with ASD as compared with those of the seven healthy controls. The total ABC scores were significantly higher in the ASD group than in the control group. The results of multiple linear regression and adaptive Lasso analyses revealed an association between increased plasma DHA levels and decreased plasma ARA levels, which were significantly associated with total ABC score and increased plasma MDA-LDL levels. Therefore, an imbalance between plasma DHA and ARA levels induces ferroptosis via lipid peroxidation. Decreased levels of α-linolenic acid and γ-linolenic acid may be connected to the total ABC scores with regard to lipid peroxidation.

## 1. Introduction

Lipids, including polyunsaturated fatty acids (PUFAs), are the main component of cell membranes and play an important role in maintaining the structural integrity of cells [1]. Lipid peroxidation is defined as a process in which oxidant-related free radicals attack polyunsaturated fatty acids (PUFAs), and it has shown its important role in cell biology and human health [1]. PUFAs produce malondialdehyde (MDA) and 4-hydroxy-2-nonenal (4-HNE) during lipid peroxidation chain reactions (Ay) [2]. MDA is used as a biomarker of lipid peroxidation [2]. Lipid peroxidation plays an important role in the pathophysiology of neurodevelopmental disorders such as autism spectrum disorder (ASD) [3]. The plasma MDA levels were significantly higher in 20 children with ASD than those in 20 age-matched controls [4], and the blood MDA levels in 45 autistic children (aged 3–11 years) were higher than those in 42 age-matched controls [5].

Potentially oxidizable PUFA species in plasma membrane samples are more abundant, and there is a need to counterbalance the antioxidant activity [6]. This can easily induce ferroptosis, leading to irreparable membrane damage and cell death [7,8]. In general, oxidants and antioxidants are driven by the activity of multiple redox-active enzymes that produce lipid oxidation products [9], with increased MDA and decreased SOD [10]. Ferroptosis is mediated by PUFA-related lipid peroxidation [11] via polyunsaturated phosphatidylethanolamines or complexes of 15-Lipoxygenase (15LOX) [12].

DHA triggers ferroptosis via the upregulation of nuclear receptor coactivator 4 (NCOA4) [8], while arachidonic acid (ARA) facilitates ferroptosis via the elongation of very long-chain fatty acid protein 5 (ELOVL5) and fatty acid desaturase-1 (FADS1) [13] or arachidonic acid 15-lipoxygenase [14]. Therefore, ferroptosis might be an important factor in lipid peroxidation in relation to DHA and ARA.

As another component of the lipid profile, low-density lipoprotein (LDL) has an important role in brain development. Of reference, significantly higher blood LDL levels were reported in 22 adults with Asperger’s syndrome (mean age, 40.8 ± 10.8 years) than in those of 22 age-matched controls, suggesting abnormal cholesterol metabolism [15]. Thus, blood LDL levels also contribute to the pathophysiology of ASD.

MDA undergoes a chemical reaction with acetaldehyde to form malondialdehyde-modified low-density lipoprotein (MDA-LDL) [15,16,17]. MDA-LDL is an important biomarker of lipid peroxidation. However, only a few studies have examined the role of MDA-LDL in the pathophysiology of ASD. This study examines the pathophysiological role of MDA-LDA in behavioral symptoms in ASD.

The antioxidant protein superoxide dismutase (SOD) is associated with MDA; for example, in a study, the lipid peroxidation and SOD levels were found to be significantly higher in 30 autistic children than they were in 30 unaffected control children [17]. However, the activity of SOD in plasma and erythrocytes is variable. For example, the serum SOD levels were increased in 20 children with ASD compared with those in 25 age-matched controls, suggesting the decreased activity of the antioxidant defense mechanism [4], while the serum SOD levels were significantly lower in autistic children aged < 6 years than they were in the age-matched controls. Additionally, the MDA level was significantly higher in these children than it was in the controls [4]. These findings suggest that children with ASD are vulnerable to oxidative stress, resulting in increased lipid peroxidation and deficient antioxidant defense mechanisms in younger children [4]. Furthermore, a close relationship between MDA and SOD was reported in 90 autistic children who were treated with coenzyme Q10 [18]. The association between MDA and SOD in the pathophysiology of ASD remains unclear. Additionally, dietary dihomo-γ-linolenic acid (DGLA; omega-6) triggers ferroptosis in the germ cells of the model organism [19]. Similarly, omega-6 PUFA adrenic acid is a lipid peroxidation product [20]. α-linolenic acid (ALA) affects lipid peroxidation due to increased oxidative stress [21]. The present study revealed the contribution of DGLA and ALA to lipid peroxidation.

Taking these considerations into account, there are two important aims of this study: (a) determining the role of DHA- and ARA-induced ferroptosis via lipid peroxidation in autistic behavioral symptoms and (b) clearly reporting the relationship between the plasma levels of MDA-LDL and SOD in individuals with autistic behaviors.

In this study, we employed the adaptive Lasso technique to ensure accurate prediction. Adaptive Lasso parameters can assist with estimation and variable selection in high-dimensional sparse models. The simulation findings and real data examples indicate the improved performance of the proposed penalized method [22]. The adaptive Lasso method is a popular statistical approach based on weighted norm penalties in weights derived from an initial estimate of a model parameter [23], and adaptive Lasso welcomes negative variable effects while specifying adaptive weights to penalize the coefficients in a different manner [23].

## 2. Materials and Methods

### 2.1. Subjects

Seventeen individuals with mild-to-moderate ASD and seven age-matched and healthy male and female control individuals were randomly included in this study. Such an imbalance in case/control individuals was recognized in a series of case–control studies, such as a case versus control study including 18 symptomatic acute intermittent porphyria patients and 33 healthy controls were performed in a recent study evaluating metabolic changes in acute intermittent porphyria patients via targeted metabolomics [24].

The information clearly presents the research methods, and the random recruitment process was conducted according to the IRB submission requirements. All 17 individuals with ASD were subjected to a medical examination at our medical clinic (Fujimoto Clinic, Kobe City, Japan), taking place between January 2020 and June 2021. Seven healthy individuals in the same region were included in this study and subjected to a medical examination at our medical clinic during the same period.

Each of the 17 individuals received a clinical diagnosis of ASD. The 17 patients with ASD included 11 males and 6 females (mean age: 11.2 ± 5.7 years). The 7 healthy controls included 4 males and 3 females (mean age: 10.0 ± 4.1 years) (Table 1). The diagnosis of ASD was conducted based on the Diagnostic and Statistical Manual of Mental Disorders, Fifth Edition (DSM-5). The Autism Diagnostic Interview-Revised (ADI-R) instrument was used to confirm the ASD diagnoses. The ADI-R interviews were conducted by one of the authors (K.Y.), who is an expert in diagnosing ASD using the Japanese version of the ADI-R. The ADI-R is a semi-structured interview conducted with the respective patients’ parents. Seventeen individuals with mild-to-moderate ASD had core symptoms of the DSM-5 diagnostic criteria for ASD without any abnormal neurological symptoms. These 17 individuals with mild-to-moderate ASD and the 8 healthy controls were matched with respect to feeding habits, age, and the full-scale intelligent quotient (Table 1). No participants had any abnormalities in the results of their physical examinations or laboratory findings. Their intelligence quotients were assessed using the Wechsler Intelligence Scale for Children and Adolescents of 6–16 years of age (WISC-V) or the respective scale for adults (Wechsler Adult Intelligence Scale (WAIC-R)). We used the Wechsler Intelligence Scale for Children, Third Edition (WISC-III), to include subjects with normal cognitive functions and a full-scale IQ of ≤70. We excluded subjects who have comorbid psychiatric illnesses using the Structured Clinical Interview for DSM-5. Individuals were excluded from this study if they had epileptic seizures or obsessive-compulsive disorder or were diagnosed with any additional psychiatric or neurological conditions. None of the individuals with ASD took dietary supplements. The healthy control subjects were recruited locally using an advertisement. All the control individuals underwent a comprehensive assessment of their medical histories to exclude individuals with neurological or other medical disorders.

### 2.2. Precautions for Mitigating the Effects of Small Sample Sizes

The small sample size in this study may limit the interpretation of the results. Therefore, we employed two precautions. First, the most appropriate method is needed with a small sample size [23]. Adaptive Lasso may select the desirable covariates to build an unbiased and statistically efficient propensity score estimator [22]. Therefore, we used adaptive Lasso. Second, standard deviations (SDs) are useful for expressing variability. Therefore, data reliability was evaluated using the coefficient of variation (CV, %), which is defined as the SD/the mean value to measure the relative variation in a random variable. The CV was employed to determine between- and within-subject reliability [25], and this is often used to measure relative variation in small sample studies.

Moreover, a large sample may not be helpful in other situations because of the higher possibility of errors and reduced validity. Analytical studies may provide more truthful results with a small sample because intensive efforts can be made to control all the confounders; small samples can be studied to reach a valid conclusion in certain situations [26]. According to a former clinical study, despite the small sample size (12 patients), statistically significant improvement was obtained by an intensive neuro-rehabilitation surface program with electromyography [27].

### 2.3. Assessment of Social Behaviors

Social behaviors were assessed using the Social Responsiveness Scale (SRS), which is used to distinguish ASD from other psychiatric disorders [28]. The SRS is a 65-item questionnaire completed by the respective parents of the subject to quantitatively assess autistic traits [28]. The SRS was used to assess the severity of the ASD symptoms [29].

### 2.4. Controlling for Dietary Intake and Assessment of Nutrient Intake

The PUFA composition of blood reflects dietary intake. In this study, all the participants received the “Japanese Food Guide” (Ministry of Health, Labour and Welfare, and Ministry of Agriculture, Forestry and Fishers, Japanese Food Guide, 2012), which is used to monitor the daily intake of nutrients and food based on the “Overview of Dietary Reference Intake for Japanese (2010)” (Ministry of Health, Labour, and Welfare, 2010). All the subjects were provided a diet based on the sample diet meal plan and menu (KAWASAKI FOODMODEL), which was edited according to the “Japanese Food Guide” (Ministry of Health, Labour and Welfare, and Ministry of Agriculture, Forestry and Fishers, Japanese Food Guide, 2012). Moreover, to measure daily food and nutrient intake, a semi-constructive questionnaire (DHQ) was performed in Japanese using a junior high school version of the DHQ15 (DHQ Support Center, http://www.ebnjapan.org/, accessed on 1 January 2020). The DHQ15 consists of 72 questions on the frequency of intake of 150 food and beverage items and cooking methods. The DHQ15 was administered for two weeks before this study. Because of the complicated assessment of DHQ15, only a subsample of 7 individuals with ASD and 7 controls participated in this assessment. Because of difficulty with nutrients in foods, the nutrient items and portion sizes in the questionnaire were derived primarily from the data in the Overview of Dietary Reference Intake for Japanese (Ministry of Health, Labour, and Welfare. Overview of dietary reference Intake for Japanese. 2015). The completed DHQ15 data sheets were checked by a psychiatrist (K.Y.). When inaccurate information was recognized, the psychiatrist (K.Y.) confirmed the data using a phone. The validity of the DHQ15 was verified. The daily intake of nutrients was calculated using a DHQ system program (DHQ support center, Tokyo, Japan).

### 2.5. Measurement of Plasma PUFA, Cp, SOD, and Tf Levels

#### 2.5.1. Blood-Sampling Procedures

Fasting exerts beneficial effects in mice and humans, including protection from chemotherapy toxicity [30]. After a 3 h fasted interval, whole blood samples were collected by venipuncture into EDTA tubes and then placed on ice. With respect to the fasting interval, the extent of return to the fasting state 2 h after a glucose challenge among 1879 normoglycemic individuals is associated with a lower risk of incident prediabetes/type 2 diabetes in the Coronary Artery Risk Development [31]. Moreover, nonfasting triglyceride levels may replace fasting levels in assessing cardiovascular disease risk once standard reference values are developed [32]. Therefore, a fasting time of 3 h after breakfast is reasonable for application for blood sampling in the present study. The blood samples were frozen at −80 °C until the plasma levels of the variables were analyzed at a clinical laboratory (SRL Inc., Tokyo, Japan).

Since the present study aimed to identify the association of lipid peroxidation with ferroptosis, we did not measure eicosanoids such as prostaglandin (PG), thromboxane (TX), and leukotrienes (LT) in the present study. The association between the levels of pro-inflammatory ARA and pro-inflammatory eicosanoids has been reported [33]; however, we did not study the association between eicosanoids and peroxidation in the present study. In response to the association between cytokine and lipid peroxidation, a few references indicated the association between proinflammatory cytokines and lipid peroxidation in patients with severe dengue disease [34].

#### 2.5.2. Plasma Levels of PUFAs

Blood samples were drawn from the 24 participants after at least 3 h of fasting. The serum specimens were separated, frozen, and stored at −80 °C until assaying. The plasma fatty acid levels were measured using the gas chromatography method (SRL, Tokyo). Twenty-four PUFAs, including the EPA, DHA, and AA concentrations, were measured. The intra- and inter-assay coefficients of ARA were 110.14 μg/mL (standard deviation (SD), 3.87; coefficient of variation (CV), 5.28%) and 100.63 μg/mL (SD, 5.51; CV, 5.48%), respectively, while those of DHA were 73.87 μg/mL (SD, 2.30; CV, 3.11%) and 68.07 μg/mL (SD, 2.30; CV, 3.33%). The plasma levels are expressed as the mean ± SD weight (%) of the total PUFAs.

#### 2.5.3. Plasma Levels of SOD

The plasma SOD levels were estimated via the cytochrome c method using an SOD Assay Kit (Takara Bio Inc., Kusatsu, Japan) in a clinical analytical laboratory (SRL Inc., Tokyo, Japan). The assay sensitivity was 0.3 U/mL. The intra- and inter-assay coefficients were 2.11 and 2.10 U/mL, respectively. (Table 1).

#### 2.5.4. Plasma Levels of CP

To estimate the plasma CP levels, a Bering BN Ⅱ Nephelometer (Siemens Healthcare Diagnostics K.K., Issaquah, WA, USA) was used. The assay sensitivity was 3.0 mg/dL.

#### 2.5.5. Plasma Levels of TF

To assess the plasma TF levels, a standard turbidimetric assay and an automated biochemical analyzer (JCA-BM8000 series, JEOL Ltd., Tokyo, Japan) were used.

### 2.6. Plasma Levels of MDA-LDL

An enzyme-linked immunosorbent assay (ELISA) was used to measure the plasma levels of MDA-LDL; the measurement was performed in a clinical analytical laboratory (SRL Inc., Tokyo, Japan). The detection limit was 6.3 U/L, and the intra- and inter-assay coefficients were <5.6% and <9.4%, respectively.

### 2.7. Sex Differences in Plasma Variables and the Total SRS Scores

To compare the plasma variables and the total SRS scores between the male and female individuals, we conducted the Mann–Whitney U test.

### 2.8. Statistical Analyses

The relationship between the plasma variables and SRS scores in the two groups was confirmed via multiple linear regression analysis (Table 2). To identify the most effective variables in small sample data, adaptive Lasso was employed. Adaptive Lasso has a good statistical performance in solving independent variables and enhancing model interpretation and prediction accuracy. Adaptive Lasso is useful for consistent variable selection and identifying important variables [22] in small samples [23]. All of the statistical analyses were performed using SPSS version 27.0.

## 3. Results

### 3.1. Study Population

Age did not significantly differ between the two groups of participants. The 17 individuals with ASD showed restricted and stereotypical behavior patterns (*n* = 10) or irritability and crying (*n* = 7). Their mean total ABC score was 49.41 ± 24.58 (Table 1). A previous study reported a total ABC score of 60.14 in 29 patients with ASD aged 13–27 years [35], Therefore, the behavioral symptoms of the 17 children in this study were moderate.

### 3.2. Plasma Levels of Lipid-Peroxidation-Related Biomarkers

**Figure 1:**
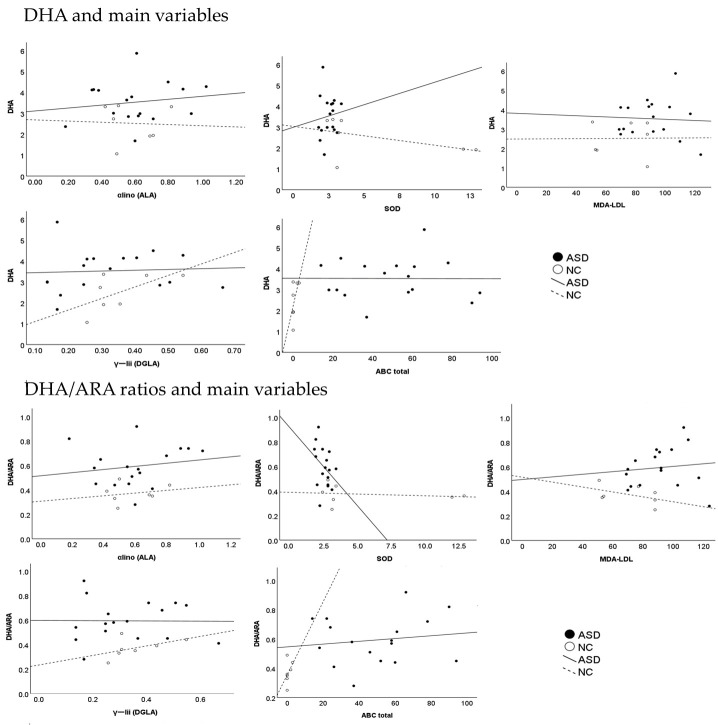
The association between the DHA and DHA/ARAS ratio and other plasma variables and the ABC.

**Figure 2:**
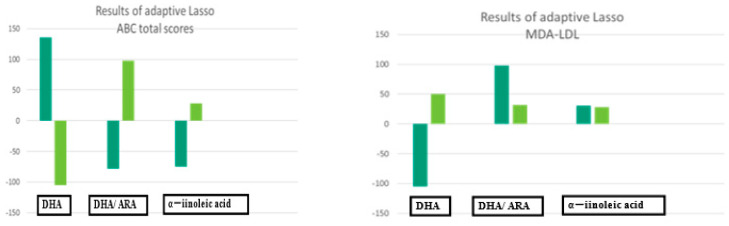
The results of adaptive Lasso.

In the ASD group, plasma MDA-LDL levels were significantly higher, and plasma SOD levels were significantly lower in comparison with the control group. The plasma DHA level and the plasma DHA/ARA ratio were significantly higher, and the plasma level of adrenic acid (AdA), and omega 6 PUFA, was significantly lower than in the control group (Table 1) (Figure 1 and Figure 2).

Plasma levels of the omega-3 PUFA DHA and the ratio of plasma DHA/ARA were significantly higher, whereas plasma levels of omega-6 family dihomo-γ-linolenic acid (DGLA) and adrenic acid (AdA) were significantly lower in the ASD group (dark green) than in the control group (light green) (Table 1).

### 3.3. Predictor Variables: Multiple Linear Regression Analysis

The multiple linear regression analysis revealed that the plasma DHA levels (R^2^ = 0.997, *p* < 0.001) and plasma DHA/ARA ratio (R^2^ = 0.982, *p* = 0.004) were significantly associated with the total ABC scores, with adjustments in plasma variables, and the total SRS scores in the two subject groups (Table 3). These findings revealed that plasma DHA levels and plasma DHA/ARA ratios may predict these variables in the two groups. Via the use of the plasma levels of α-linolenic acid (ALA) as the dependent variable, these levels showed the significant contribution made by the plasma DHA levels (unstandardized coefficients, *B* = 0.849 ± 0.245 *β* = 0.1641, *p* = 0.02) and the plasma ratios of DHA/ARA (unstandardized coefficients, *B* = −0.342 ± 0.124 *β* = −0.395, *p* = 0.041) (Table 2) (Figure 1). Therefore, the plasma DHA levels and plasma DHA/ARA ratio distinguished the ASD group from the control group.

### 3.4. Results of Adaptive Lasso

We employed the adaptive Lasso approach to increase the predictive accuracy and interpretability of the statistical model for small samples [20,21]. The plasma levels of DHA (standard coefficient, 136.50; 95% CI, 29.10 to 243.90; *p* = 0.017) and the plasma ratios of ARA (coefficient, −104.84; 95% CI, 193.69 to 25.98 *p* = 0.000) and ALA (coefficient, −75.48) were selected for the ABC total scores (Table 3) (Figure 2).

### 3.5. The Profiles of Nutrient Intake

The intake of nutrients in the random subsamples of 7 of the 17 individuals and 5 of the 7 normal controls were analyzed, and there were no significant differences in the nutrient intake profile between both subsamples (Table 4).

### 3.6. Sex Difference

There were no gender differences in plasma levels of the main variables (Table 5).

## 4. Discussion

Two measures were taken in this study because of the small sample size. The mean CVs for the plasma PUFAs were 0.451 (45.17%) and 0.277 (27.7%) in the ASD and control groups, respectively. With respect to the CVs, the plasma levels of the anti-cancer drug palbociclib were variable, with a CV from 38.8% to 48.5% [36]. Furthermore, CV in cardiothoracic ratio on chest radiograph in pediatric heart disease was 32.5% Gro [37]. Thus, the CVs in the present study were comparable to these previously reported CVs, suggesting the appropriateness of our variable selection.

In the present study, in the ASD group, the plasma MDA-LDL levels were significantly higher, and the plasma SOD levels were significantly lower in comparison with those of the control group. The plasma DHA level and the plasma DHA/ARA ratio were significantly higher in the 17 subjects with ASD than they were in the control group (Table 1). Additionally, the omega-3 PUFA family AdA and omega-6 PUFA family GLA levels in the plasma were more significantly decreased in the ASD group as compared with those of the control group. Notably, the results of multiple linear regression analysis and the adaptive Lasso method revealed that increased DHA and decreased ARA plasma levels were appropriate variables that distinguished the ASD group from the normal control group. Importantly, higher plasma MDA-LDL levels in the ASD group indicated ferroptosis [9,10]. PUFAs are highly susceptible to lipid peroxidation under oxidative stress [7]. The lipid peroxidation of cellular membranes caused by the disruption of the antioxidant defense system induces ferroptosis, which is a type of regulated cell death. Ferroptosis is a recently proposed novel mode of cell death. The inhibition of ferroptosis may provide the possibility to prevent and improve impaired neurocognitive disorders [38].

DHA and EPA prevent seizure and depression-like behavior by inhibiting ferroptosis and neuroinflammation [39]. DHA is sensitized to ferroptosis via an increase in ROS accumulation and lipid peroxidation (Shan) [40]. Omega-6 PUFAs have relatively greater susceptibility to lipid peroxidation and can participate in ferroptosis [7]. In a study, the downregulation of arachidonate 5-lipoxygenase (ALOX5) inhibited ferroptosis, while the overexpression of ALOX5 promoted ferroptosis [40]. ARA associated with endoplasmic reticulum phospholipids induces ferroptosis due to the interaction with T-cell-generated interferon, contributing to death by ferroptosis [7]. Omega-3 PUFAs promote intracellular antioxidant synthesis and reduce the formation of hydroperoxides, inducing ferroptosis [41]. DHA is sensitized to ferroptosis via an increase in ROS accumulation and lipid peroxidation [7].

Omega-6 PUFAs have relatively greater susceptibility to lipid peroxidation and can participate in ferroptosis [7]. In a study, the downregulation of arachidonate 5-lipoxygenase (ALOX5) inhibited ferroptosis, while the overexpression of ALOX5 promoted ferroptosis [7]. ARA associated with endoplasmic reticulum phospholipids induces ferroptosis due to the interaction with T-cell-generated interferon, contributing to death by ferroptosis [7].

Furthermore, ARA transport may be an important contributor to ferroptosis sensitivity (Dier). These previously reported findings support the present findings in that increased DHA levels and decreased ARA levels in plasma may induce ferroptosis in the ASD group.

The present study included decreased plasma levels of ALA and DGL in the ASD groups. ALA can induce ferroptosis via an isomer, α-eleostearic acid [42]. Based on the accumulated evidence, the ACSL4-catalyzed biosynthesis of arachidonoyl-CoA contributes to the execution of ferroptosis via triggering phospholipid peroxidation [12]. DGL-related ferroptosis is a complex area of research, but there have been studies of GLA metabolism and its relationship with inflammatory processes, including ferroptosis [43]. DGLA induces ferroptosis-mediated neurodegeneration in dopaminergic neurons, representing a new class of lipid metabolite that induces neurodegeneration via ferroptosis [44].

Regarding the effects of the combination of DHA and ARA on lipid peroxidation, a previous study found that both DHA and ARA are susceptible to peroxidation by oxygen free radicals, producing 4-hydroxynonenal (4-HNE) from ARA via 12-hydroperoxy-eicosatetraenoate and 4-hydroxyhexenal (4-HHE) and from DHA via the deacylation–reacylation cycle [45]. Thus, these results regarding the contribution of both plasma DHA and ARA levels to lipid peroxidation are reasonable. DHA promotes synaptic connectivity and the prospective therapeutic capacity for neurodegenerative diseases [46]. DHA’s antioxidant activity plays an important role in signaling and lipid mediator production [47]. Furthermore, DHA has an important role in synaptogenesis and the synaptic expression of synapsin, which exerts beneficial effects on neuronal plasticity [48] and modulates the oxidant/antioxidant balance by increasing the SOD level and decreasing the MDA-LDL level [49]. Therefore, increased plasma DHA levels may play an important role in synaptic function and in regulating redox homeostasis, thus supporting our findings. However, the intake of high doses of DHA can promote lipid peroxidation [47], possibly due to cellular damage caused by oxidation [50]. Therefore, the incorporation of DHA may enhance people’s susceptibility to lipid peroxidation and disrupt the antioxidant system. These previous findings may indicate sensitivity to lipid peroxidation via DHA, as shown in this study.

The release of ARA induces lipid peroxidation (4-HNE) via cytosolic phospholipase A_2_α (cPLA_2_α), making ARA an important lipid signal in activated immune cells [51], while ARA is metabolized to prostaglandin E2 (PGE2), and both abnormal PGE2 signaling and Wnt signaling contribute to ASD [52]. Therefore, ARA plays an important role in lipid peroxidation in relation to the development of ASD. In addition, with respect to differences in the activities of lipid peroxidation between DHA and ARA, the activity of DHA may be due to an increase in DHA-related 4-HHE due to the Nrf2/antioxidant response element system, whereas ARA-related 4-HNE activity depends mainly on the stimulation of cells with lipopolysaccharides related to the increase in cPLA2 [45]. The metabolic link between DHA and the 4-HHE pathway may be useful in therapeutic strategies against oxidative damage due to cerebral ischemia and other brain injuries [40]. Furthermore, upon exceeding the buffering capacity of triglyceride storage into lipid droplets, both omega-3 and omega-6 PUFAs induce peroxidation, leading to cytotoxic effects in the presence of diacylglycerol acyltransferase inhibitors (DGATis) [41]. Therefore, the present finding that both DHA and ARA contribute to lipid peroxidation may be reasonable. The decreased plasma levels of ALA may be involved in both DHA and ARA lipid peroxidation.

The DHA/ARA balance is important in the cognitive and behavioral development of infants [53]. Our previous study revealed that an increased plasma omega-3/omega-6 ratio due to decreased plasma ARA levels results in a decrease in plasma levels of signaling proteins, such as ceruloplasmin, and these mechanisms contribute to the pathophysiology of ASD [54]. An omega-6/omega-3 PUFA ratio of 4:1 is advocated because of competition for the same enzymes during desaturation and elongation and the protective and stabilizing capacity of the neuronal membrane (Yahuda) [55]. Values of 17 mg/100 kcal for DHA and 34 or 25 mg/100 kcal for ARA and (DHA/ARA ratio, 17/25, 2.3/1) advocate for the development of the nervous system of infants [53]. Importantly, the reported beneficial ratio of DHA/ARA is at least 2:1.5 [53]. Therefore, an imbalance between plasma DHA and ARA levels might induce autistic behavioral symptoms and altered plasma MDA-LDL levels. The average DHA/ARA ratio in the ASD group in the present study was 0.59 (1:1.9), which might have been non-beneficial.

DHA is synthesized from ALA via ELOVL Fatty Acid Elongase 2 (ELOVL2), a rate-limiting enzyme of DHA synthesis in cells [56]. Therefore, the increase in plasma DHA levels in the present study may reflect a comparable increase in the activity of ELOVL2. Moreover, since the metabolism of omega-3 PUFA are synthesized by delta-12 and delta-15 desaturase enzymes [57], this enzyme may be lowered, resulting in low DAH levels.

The plasma levels of omega-6 PUFA family members DGLA and adrenic acid (AdA)-induced oxidative stress blocked serotonin synthesis and serotoninergic neuron activity [58]. In the present study, the AdA level was significantly lower in the ASD group than it was in the control group. AdA upregulates the production of intracellular oxidative-stress-related ROS. Therefore, reduced plasma AdA levels may indicate weak anti-oxidative stress activity and may contribute to autistic behaviors in ASD individuals, as shown in the present findings. The plasma levels of omega-6 PUFA family member DGLA can be further converted to prostaglandin E1, which possesses anti-inflammatory properties [59]. The anti-inflammatory effects of DGLA downregulate the synthesis of lipid mediators of inflammation, thereby contributing to the improvement in clinical symptoms of inflammatory disorders such as ASD [60]. DGLA can be further converted to prostaglandin E1, which possesses anti-inflammatory properties [60]. Taken together, reduced plasma DGLA in the present study may weaken the anti-inflammatory activity. Overall, reduced plasma AdA and DGLA levels may weaken antioxidant and anti-inflammatory activities, contributing to autistic social behaviors.

MDA is an endogenous genotoxic product of enzymatic and oxygen-radical-induced lipid peroxidation [45]. DHA secondarily elicits MDA production [61]. Therefore, plasma MDA-LDL, as the end-product of lipid peroxidation, might have had a small, indirect effect on the behavioral symptoms of the ASD individuals in our study.

In the present study, the plasma SOD levels were significantly lower in the ASD group than they were in the control group. SOD controls oxidative damage and regulates signaling activities [62]. The loss of SOD2 activity may, therefore, result in various pathological phenotypes in the central nervous system [63]. We previously reported that reduced plasma SOD levels may be related to the decreased endogenous antioxidant capacity [63]. Therefore, decreased plasma SOD levels may contribute to the neuronal deficit related to behavioral symptoms in autistic individuals.

Several limitations associated with the present study warrant mention. First, 4-HNE, as the main phospholipid product, is considered a second messenger of ROS, which is an important factor in stress- and age-associated diseases [64]. 4-HNE plays an important role in not only physiological and protective functions as a signaling molecule but also plays a cytotoxic role in inhibiting gene expression and promoting cell death [2]. Therefore, we used the plasma levels of MDA-LDL as a biomarker of lipid peroxidation. Second, the small sample size limits the generalization of these results to the entire ASD population. Importantly, we applied two useful measures to ameliorate the difficulty in drawing significant conclusions. A previous review article indicated similar results to our findings concerning the association between MDA and SOD [4,64]. Furthermore, many previous studies have indicated that DHA/ARA ratios are important for lipid peroxidation [65,66,67]. Third, the ratio of control subjects to cases was small (2.3:1), and the average DHA/ARA ratio in the ASD group in the present study was 0.59 (2:3.79). A previous case–control genomic study found that ratios of 3:1 or 4:1 induced more significant results compared with 1:1 or 2:1 [68]. This ratio of the experimental group to the control group was 2:1 in a previously randomized clinical trial of a new drug [69]. Importantly, the most beneficial ratio of DHA/ARA was reported to be at least 2:1.5 [53]. The average DHA/ARA ratio in the ASD group in the present study was 0.59 (2:3.79), which might have been non-beneficial.

ALA may protect against mercurial-induced abnormal social interactions and stereotypical behaviors in mice [70]. Similarly, ALA has a protective role against valproic-acid-induced autism-like features [71]. Thus, decreased ALA and GLA levels may be associated with the total ABC scores.

DHA-ARA balance is important in cognitive and behavioral development in infants [53]. Our previous study revealed that an increased plasma omega-3/omega-6 ratio reflects decreased plasma ARA levels, resulting in decreases in the plasma levels of signaling proteins such as ceruloplasmin and that this ratio may contribute to the pathophysiology of ASD [63]. An omega-6/omega-3 PUFA ratio of 4:1 is advocated because of competition for the same enzymes during desaturation and elongation and the protective and stabilizing capacity of the neuronal [68]. ARA:DHA ratio greater than 1:1 is associated with improved cognitive outcomes [61]. Therefore, an imbalance between plasma DHA and ARA levels might contribute to autistic behavioral symptoms. Importantly, while the reported beneficial ratio of DHA/ARA is at least 2:1.5 [53], the average DHA/ARA ratio in the ASD group in the present study was 0.59 (2:3.79), which might have been unbeneficial.

DHA is synthesized from ALA in the body via ELOVL fatty acid elongase 2 (ELOVL2), a rate-limiting enzyme of DHA synthesis in cells [72]. Therefore, our study suggests for the first time that increased plasma DHA levels may reflect a comparable increase in the activity of ELOVL2 and an insufficient supply of ALA and then induce an unbeneficial DHA/RA ratio.

The plasma levels of omega-6 PUFA family member DGLA were significantly lower in the ASD group than in the control group. The anti-inflammatory effects of dihomo-γ-linolenic acid (DGLA) downregulate the synthesis of lipid mediators of inflammation [73], thereby contributing to the improvement in the clinical symptoms of inflammatory disorders such as ASD [63].

Several limitations associated with the present study warrant mention. First, 4-HNE, as the main phospholipid product, is considered a second messenger of ROS [45]. At the same time, MDA is an endogenous genotoxic product of enzymatic and oxygen-radical-induced lipid peroxidation [74]. Since MDA is a biomarker of the lipid peroxidation of n-3 and n-6 PUFAs [2], we used plasma levels of MDA-LDL but not 4-HNE. Second, the small sample size limits the generalization of these results to the entire ASD population. Importantly, we applied two measures to ameliorate the difficulty in drawing significant conclusions. A previous review article indicated similar results concerning the association between MDA and SOD [4,17]. Furthermore, many previous studies have indicated that DHA/ARA ratios are important for lipid peroxidation [53,54,55]. Third, the ratio of control to case subjects was small (2.3:1), and the average DHA/ARA ratio in the ASD group in the present study was 0.59. A previous case–control genomic study found that ratios of 3:1 or 4:1 induced more significant results compared with 1:1 or 2:1 [53]. This ratio of the experimental group to the control group was 2:1 in a previous randomized clinical trial of a new drug [53]. Importantly, the beneficial ratio of DHA/ARA was reported to be at least 2:1.5 [53]. The average DHA/ARA ratio in the ASD group in the present study was 0.59 (2:3.79), which might have been non-beneficial.

## 5. Conclusions

These multiple linear regression analysis and adaptive Lasso analysis results revealed that the concomitance of increased plasma DHA levels and decreased plasma ARA levels may induce increased plasma MDA-LDL levels. This imbalance between DHA and ARA levels in plasma induced ferroptosis via lipid peroxidation in patients with autistic behavioral symptoms. The disturbed metabolism of decreased levels of ALA subsequently induced decreased levels of GLA and may have induced oxidation with regard to lipid peroxidation. This biomedical mechanism may be due to the rate-limiting enzyme of DHA synthesis, cell elongase 2 (ELOVL2). These neurological impairments may contribute to autistic behavioral symptoms.

## Figures and Tables

**Table 1:** Subject characteristics and plasma levels of PUFAS, MDA-LDL, and antioxidant proteins, and ABC total scores.

	ASD	Controls	U	*p* Value
	(n = 17)	(n = 7)		
Age (years)	11.2 ± 5.7	10.0 ± 4.1	45.00	0.49
Sex (male/female)	13/17	4/17	χ^2^ = 0.46	0.50
Scores of Autism Diagnostic				
Interview-revised				
Domain A (Social)	13.6 ± 4.3			
Domain B (Communication)	2.1 ± 2.3			
Domain C (stereotyped)	17.6 ± 5.6			
Plasma biomarker levels				
Cp (mg/dL)	28.29 ± 7.21	24.29 ± 7.25	44.50	0.35
Tf (mg/dL)	275.71 ± 43.16	262.29 ± 25.75	47.00	0.46
SOD (mg/dL)	2.51 ± 0.47	5.69 ± 4.64	18.50	0.01 *
MDA-LDL	90.94 ± 17.24	71.29 ± 17.87	24.50	0.02 *
DHA	3.53 ± 0.98	2.52 ± 0.90	28.50	0.047 *
ARA	6.34 ±1.11	6.68 ±1.67	49.00	0.53
DHA/ARA	0.59 ± 0.17	0.37 ± 0.08	11.50	0.01 *
AdA	0.27 ± 0.25	0.27 ± 0.07	28.50	0.047 *
DGLA	1.27 ± 0.33	1.60 ± 0.22	25.50	0.03 *
Total scores of Aberrant Behavior Checklist	49.41 ± 25.57	0.71 ± 1.25	0.00	*p* < 0.001 **

Cp, ceruloplasmin; Tf, transferrin; SOD, superoxide dismutase; MDA-LDL, malondialdehyde modified low-density lipoprotein; DHA, docosahexaenoic acid; ARA, arachidonic acid; DGLA, dihomo-γ-linolenic acid: AdA, adrenic acid; ABC, Aberrant Behavior Checklist. Values are represented as mean ± SD (Mann–Whitney U test). * *p* < 0.05. ** *p* < 0.001, versus normal controls.

**Table 2:** Results of the multiple linear regression.

Model	Model R^2^	Model Value	Coefficients		
			B	Beta Coefficients	*p* Value
DHA	0.997	*p* < 0.001 **			
ALA			0.849 0.245	0.164	0.018
DGLA			−0.210 0.029	−0.421	0.002
MDA-LDL			−0.057 0.124	−0.025	0.667
SOD			−0.0076 0.020	−0.203	0.012
ABC total scores			0.002 0.002	0.067	0.253
Group 1 = ASD, 2 = controls			−0.057 0.124	−0.025	0.667
DHA/ARA	0.982	0.004 **			
ALA			−0.342 0.124	−0.395	0.041
DGLA			−0.122 0.058	−0.23-	0.090
MDA-LDL			0.002 0.001	0.270	0.096
SOD			0.008 0.050	0.023	0.895
ABC total scores			0.022 0.024	0.348	0.183
Group 1 = ASD, 2 = control			0.007 0.055	0.019	0.899

R^2^, R-squared values; B, Unstandardized coefficients; ALA, α-linolenic acid; DGLA, dihomo-γ-linolenic acid; SOD, superoxide dismutase; MDA-LDL, malondialdehyde-modified low-density lipoprotein; ABC, Aberrant Behavior Checklist. ** *p* < 0.001.

**Table 3:** Results of adaptive Lasso.

Intercept	Estimate	SE	*p* Value	95%	Confidence Interval
				Lower Bounds	Upper Bounds
ABC total scores					
DHA	136.50	54.89	0.012	29.11	243.90
DHA/ARA	−74.88	46.97	0.111	−166.94	17.18
α-linoleic acid	−75.48	26.21	0.004	−126.84	−24.12
MDA-LDL					
DHA	−105.88	50.74	0.038	−205.32	−6.44
DHA/ARA	98.81	32.21	0.002	35.67	161.95
α-linoleic acid	31.38	28.01	0.247	−22.52	87.28

**Table 4:** The intake of nutrients in the random subsamples of 7 of the 17 individuals and 5 of the 7 normal controls.

	ASD (n = 7)	Control (n = 5)	U	*p* Value
Age (years)	11.4 ± 4.3	11.4 ± 3.2	14.0	0.87
Fat (g/day)	72.2 ± 30.1	87.4 ± 25.8	12.0	0.43
Unsaturated fatty acid (g/day)	14.8 ± 4.4	18.7 ± 5.5	9.0	0.20
Omega-3 PUFAs (g/day)	2.6 ± 0.8	3.1 ± 0.5	9.0	0.20
Omega-6 PUFAs (g/day)	12.1 ± 3.9	15.9 ± 5.1	11.0	0.34
EPA (mg/day)	181.2 ± 118.7	176.2 ± 73.6	15.5	0.76
DHA (mg/day)	332.6 ± 170.4	345.0 ± 83.31	15.5	0.76
ARA (mg/day)	168.1 ± 17.1	221.0 ± 87.7	11.5	0.34
Protein (g/day)	78.1 ± 25.8	89.2 ± 25.8	13.0	0.53
Animal protein (mg/day)	32.0 ± 9.1	30.4 ± 14.7	14.0	0.64
Cholesterol (mg/day)	139.1± 186.4	31.9 ± 10.3	17.0	1.00
Carbohydrates (g/day)	286.2 ± 62.1	304.21 ± 72.4	14.0	0.64

EPA, eicosapentaenoic acid; DHA, docosahexaenoic acid; ARA, arachidonic acid. Values are mean ± SD.

**Table 5:** Sex differences in plasma variables and the total SRS scores.

	MDA-LDL	DHA	ARA	SOD	Total ABC Score
Male (n = 12)	92.55 ± 14.69	3.43 ± 1.07	6.43 ± 1.12	2.76 ± 0.53	41.81 ± 29.03
Female (n = 5)	88.93 ± 29.88	3.05 ± 0.71	6.32 ± 1.77	4.21 ± 3.54	19.78 ± 29.68
U	112.00	100.50	139.00	99.00	45.50
*p* values	0.34	0.17	0.99	0.16	0.07

DHA, docosahexaenoic acid; ARA, arachidonic acid; SOD, superoxide dismutase; ABC, Aberrant Behavior Checklist.

## Data Availability

Corresponding author (Kunio Yui) may response to requires.
